# Safety and Efficacy of Combined Yttrium 90 Resin Radioembolization with Aflibercept and FOLFIRI in a Patient with Metastatic Colorectal Cancer

**DOI:** 10.1155/2015/461823

**Published:** 2015-03-16

**Authors:** Andre De Souza, Kevin Pelham Daly, James Yoo, Muhammad Wasif Saif

**Affiliations:** ^1^Hematology/Oncology Department, Tufts Medical Center, 800 Washington Street, Boston, MA 02111, USA; ^2^Interventional Radiology Department, Tufts Medical Center, 800 Washington Street, Boston, MA 02111, USA; ^3^Surgery Department, Tufts Medical Center, 800 Washington Street, Boston, MA 02111, USA

## Abstract

*Background*. When associated with isolated four or fewer liver foci, metastatic colorectal cancer is amenable to surgical resection. Alternative therapeutic methods for isolated liver metastases include radioembolization with yttrium 90 (Y90) and transarterial chemoembolization (TACE). We present here a case of a patient with two sites of liver metastatic disease from colorectal cancer who underwent Y90 radioembolization combined with aflibercept and FOLFIRI. *Case Report*. A 56-year-old female with history of bilateral breast cancer and metastatic colon cancer with prior hemicolectomy and 4 previous chemotherapy regimens developed liver metastasis. She was started on aflibercept and FOLFIRI and concurrently underwent two treatments of radioembolization with Y90, initially targeting the largest right lobe tumor, and then a subsequent treatment targeting the smaller left lobe tumor with retreatment of the right lobe tumor. Her liver metastases exhibited partial response on imaging utilizing the modified RECIST criteria. Interestingly, the patient CEA levels decreased after the procedure. *Discussion*. This is the first reported case of a patient managed with radioembolization with Y90 combined with aflibercept, an anti-VEGF treatment, and FOLFIRI. An ongoing randomized clinical trial aims to define the role of combined targeted therapy and chemotherapy with radioembolization with Y90.

## 1. Introduction

Colorectal cancer is the fourth most prevalent cancer and the second leading cause of cancer-related mortality in the United States [1]. About 50–60% of the colorectal cancers are expected to metastasize [2]. Around 50% of the patients with colorectal cancer will develop liver metastases during the course of their disease and 25% will have liver metastases on presentation. From the group of patients with liver metastases, one-third will have isolated hepatic metastases. Patients with liver metastases have a median overall survival of around 4 months.

Patients with liver metastases are part of the larger group of metastatic colorectal cancer and will have indication to chemotherapy if they are not isolated metastases. First-line chemotherapy for Stage IV disease usually involves 5-fluorouracil and leucovorin combined with either oxaliplatin (FOLFOX) or irinotecan (FOLFIRI); capecitabine associated with oxaliplatin (XELOX) is also an option [3]. Presence of K-ras wild type gene predicts response to epidermal growth factor receptor (EGFR) monoclonal antibodies such as cetuximab or panitumumab, and they are usually combined with FOLFIRI (or FOLFOX in the case of panitumumab) in the treatment of metastatic colorectal cancer [4, 5]. Patients with Stage IV colorectal cancer with K-ras mutation may benefit from VEGF inhibitors such as bevacizumab or aflibercept [6, 7]. This effect was not shown with tyrosine kinase inhibitors and antiangiogenic agents such as sorafenib [8]. The multikinase inhibitor regorafenib has been indicated by the FDA as a third-line agent for metastatic colon cancer [9]. A multicenter randomized double-blind trial from 2012 has proven that aflibercept, a soluble VEGF receptor, improves overall survival when added to FOLFIRI (5-fluorouracil, leucovorin, and irinotecan) in metastatic colorectal cancer. This benefit extended even for patients who were previously on bevacizumab, another VEGF inhibitor. The authors reported 1.44-month improvement in median overall survival for patients on FOLFIRI who received aflibercept as opposed to placebo (*P* = 0.0032) [7].

Patients with four or fewer isolated liver metastases benefit from regional therapy. These include surgical resection, radiation therapy, local tumor ablation by alcohol, acetic acid or radiofrequency ablation, TACE, and radioembolization [10–12]. Traditionally, only surgery was associated with improved survival. Five-year survival rate from surgery can reach up to 40%. One-third of the five-year cancer survivors will have a cancer-specific death. Around 17% will achieve ten-year survival. However, only 20% of the patients with isolated liver metastases are able to undergo curative resection. Neoadjuvant chemotherapy is indicated for nonresectable disease or borderline resectable disease that is potentially convertible to resectable disease. Response should be assessed in six-week intervals and chemotherapy should be completed 4 weeks before the surgical resection and six to eight weeks if bevacizumab is given. TACE had mixed results from the two randomized clinical trials available in the literature [12, 13].

Cianni et al. have investigated over 3 years the safety and toxicity profile of Y90 radioembolization to liver metastases from colorectal cancer that are not responsive to chemotherapy. Stable disease was achieved in 33% of the patient while response rates benefited 50% of the patients [13]. In a systematic review by Saxena et al. comprising 979 patients, complete response, partial response, and stable disease were 0% (range 0–6%), 31% (range 0–73%), and 40.5% (range 17–76%), respectively. The median time to liver metastasis progression was 9 months (range 6–16). The median overall survival was 12 months (range 8.3–36) [14]. In a study by Rafi et al. involving 19 patients, fatigue and transient abdominal pain were the main adverse effects, observed in 4 (21%) and 6 (32%) patients, respectively [15].

## 2. Case Report 

Although data on the combination of chemotherapy and radioembolization with yttrium 90 microspheres is available from small case series, safety and efficacy with the concomitant use of anti-VEGF therapy are still not available. We herein present the first case in the literature on such a regimen. A 56-year-old female with history of bilateral breast cancer and Stage IV (T3N2M1) colon cancer with liver and lung metastases diagnosed in July 2012 with prior left hemicolectomy and oophorectomy for breast cancer risk reduction presented to our service in June 2013.

The patient had a history of breast cancer diagnosed in October 2008 treated with neoadjuvant doxorubicin and cyclophosphamide regimen followed by paclitaxel. She had right-sided T2 disease with one lymph node positive. In the left side she had N2 disease with 3 out of 17 lymph nodes positive for metastasis, extracapsular extension, estrogen and progesterone receptor positive, and Her/neu negative. Due to extracapsular extension, she underwent bilateral mastectomy and adjuvant bilateral breast irradiation. Also, as she had hormone receptor status positive, she was started on leuprolide and anastrozole in the fall of 2008.

**Figure 1:**
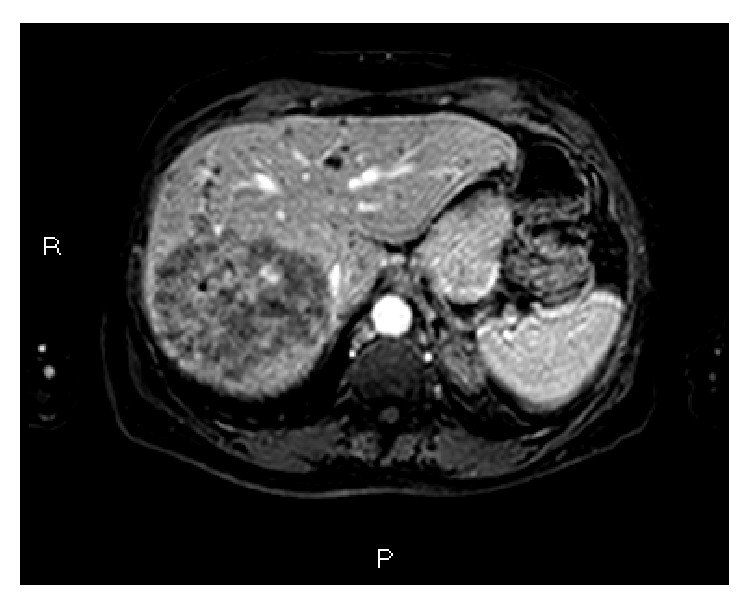
Gadolinium enhanced T1 weighted MRI of the abdomen with fat saturation prior to treatment with radioembolization shows right hepatic lobe with 8 cm × 9 cm mass (06/07/2013). Enhancement within the tumor indicates tumor viability.

**Figure 2 fig2:**
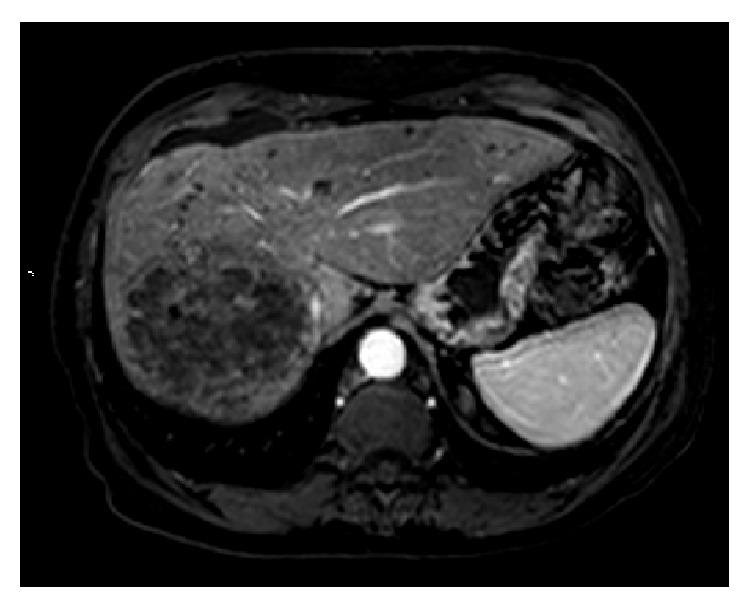
Same lesion on MRI 2 months following radioembolization with yttrium 90 resin microspheres (09/20/2013). There is no enhancement within the central portion of the mass and the tumor has decreased slightly in size, consistent with partial response by mRECIST criteria.

**Figure 3:**
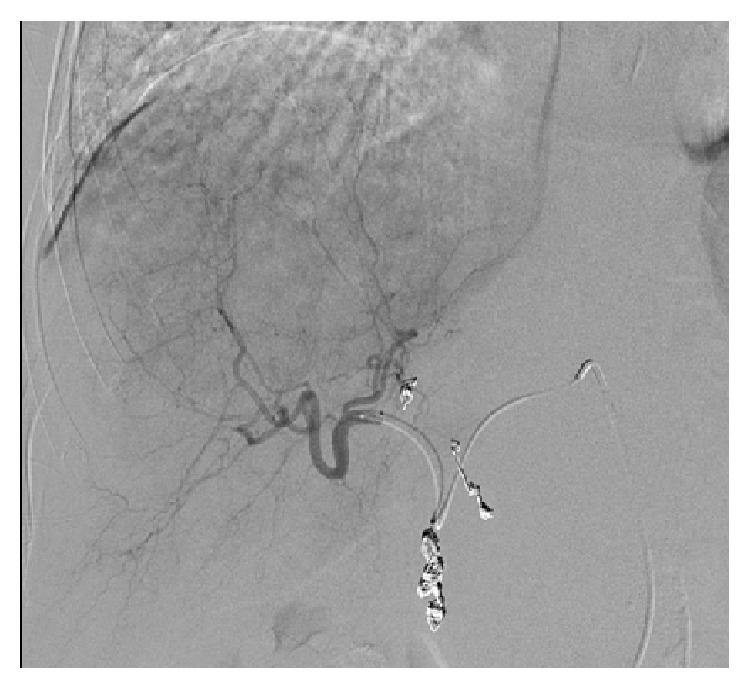
Angiography (08/08/2013) of right hepatic lobe lesion before Y90 treatment showing extensive tumor vascularity of the dominant right lobe lesion being fed by branches of the right hepatic artery. Coil embolization of gastroduodenal artery and right gastric artery had been previously performed to prevent nontargeted delivery of microspheres to the gastrointestinal tract.

**Figure 4:**
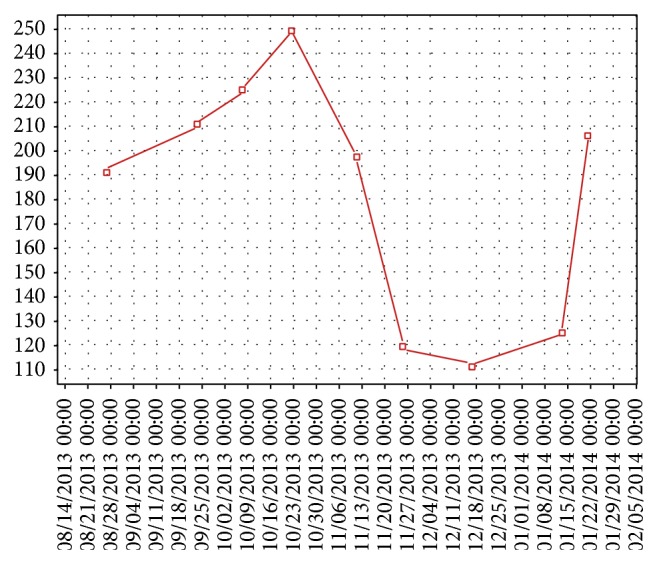
CEA trend from August 2013 to February 2014.

The patient has been on previous chemotherapy regimens for Stage IV colon cancer including XELOX (capecitabine and oxaliplatin), FOLFOX (5-fluorouracil, leucovorin, and oxaliplatin), Xeloda-bevacizumab (capecitabine and bevacizumab), and XELOX-bevacizumab (capecitabine, oxaliplatin, and bevacizumab).  Table 1 summarizes the treatment course and the radiologic assessment since her care was started in our service. A baseline MRI showed 3 liver metastases, with largest lesion in the right lobe measuring 9.4 cm in the largest diameter. She was started on aflibercept and FOLFIRI (5-fluorouracil, leucovorin, and irinotecan) repeated every 2 weeks, with the first cycle on 06/17/2013, receiving intravenously aflibercept = 4 mg/Kg, irinotecan = 150 mg/m^2^, leucovorin = 100 mg/m^2^, and fluorouracil = 2000 mg/m^2^ [16]. After the first cycle the patient underwent radioembolization with yttrium 90 resin microspheres targeting the largest right lobe hepatic tumor (07/03/13). The second chemotherapy cycle was held until 07/15/13 due to the increased risk of bleeding on anti-VEGF treatment. A second session of Y90 radioembolization targeting both hepatic lobes was performed on 08/8/2013. A CT scan of the abdomen in July 2013 prior to the radioembolization procedure showed an 8 cm × 9 cm right lobe hepatic mass, as shown in Figure 1. MRI of the abdomen following radioembolization treatment in September showed decrease in the overall size of the dominant right lobe mass and decreased enhancement within the masses, indicative of a partial response according to modified RECIST criteria (Figures 1 [Fig fig2]–3) [17]. After developing fatigue and dehydration from 5-fluorouracil treatment, her chemotherapy was changed from aflibercept and FOLFIRI to aflibercept and irinotecan on the 8th cycle (11/21/2013). A total of 11 cycles of chemotherapy were completed. Our patient had a good response after the procedure, as shown by CEA levels.  Figure 4 shows the decrease in CEA levels while the patient was in our service. A drop in the CEA level was noticed as of October 2013 with nadir in December 2013. Of note, there was no change in the chemotherapy regimen from June to November 2013. Unfortunately, our patient's CEA levels are again increasing and follow-up imaging showed progression of lung metastases but stable disease within the liver. Additionally, abdominal imaging on 12/30/2013 showed interval development of small abdominal ascites (from trace on prior imaging) and slightly nodular contour to the liver, reflecting treatment related effects of prior radioembolization and chemotherapy. Liver function tests remained within the normal range with no other evidence of liver decompensation. As she had progression of her disease with increased tumor burden in the lungs, she was started on an experimental agent targeting VEGF-3 receptor on 01/21/2014.

## 3. Discussion

Yttrium 90 microsphere radioembolization is a selective internal radiation therapy based on the concept of regional therapy for isolated liver metastasis in metastatic colorectal cancer. Although studies have shown good outcomes from combination chemotherapy associated with yttrium 90 microsphere radioembolization, we report the first case of aflibercept combined with FOLFIRI and yttrium 90 radioembolization in metastatic colorectal cancer. We believe our case revealed safety as well as efficacy as liver metastases remained in partial response even at the end of five months as well as declining CEA.

A phase II trial (the AFFIRM study) evaluating the safety and efficacy of aflibercept when added to FOLFOX has shown a similar overall response rate and progression free survival to FOLFOX alone in patients with metastatic colorectal cancer [18]. When a phase III trial (the VELOUR study) was devised to compare FOLFIRI and aflibercept versus FOLFIRI plus placebo in metastatic colorectal cancer after oxaliplatin failure, improved median overall survival (13.50 months for aflibercept and 12.06 months for placebo; HR = 0.817, *P* = 0.0032), progression free survival (6.90 months versus 4.67 months; HR = 0.758, *P* = 0.0001), and overall response rate (19.8% versus 11.1% in the placebo, *P* = 0.001) were found. A trend towards improved overall survival and progression free survival was achieved even when the patients were previously on bevacizumab [7].

Cosimelli et al. investigated the safety and efficacy of Y90 radioembolization in colorectal cancer metastatic to the liver. Although these patients had worse prognostic features than the previous studies, having chemotherapy refractory disease to at least 4 regimens, they achieved significant response (both overall response rate and stable disease were 24%) [19]. Sharma et al. investigated the safety of first-line FOLFOX4 regimen associated with Y90 microsphere radioembolization in colorectal cancer with metastasis to the liver [20]. Seidensticker et al. designed a phase III randomized clinical trial to define the role of Y90 radioembolization in colorectal cancer with liver metastasis. With a population of 29 patients receiving Y90 radioembolization matched with a 500 population of patients on best supportive care (BSC), the goal of the study was to evaluate overall survival as the primary end point. Compared with BSC alone, radioembolization prolonged median overall survival (8.3 versus 3.5 months; *P* < 0.001) [21]. The same group also investigated a sequential approach using selective lobar radioembolization that showed less hepatotoxicity than whole liver radioembolization, with less liver-related grade 3/4 adverse effects (14 versus 2 patients, *P* < 0.05) and pathological increases in bilirubin at 3 months (52.3 versus 18.7 *μ*mol/L, *P* = 0.012) [22]. Hendlisz et al. compared the effect of protracted 5-fluorouracil (5-FU) infusion versus Y90 radioembolization and protracted 5-FU infusion in a phase III randomized trial. The primary end point was time to liver progression, which was significantly longer in the Y90 arm (5.5 months versus 2.1 months, hazard ratio 0.38, *P* < 0.001) [23]. Although there was no statistically significant difference in median overall survival (7.3 versus 10.0 months in arms A and B, respectively (HR = 0.92; 95% CI, 0.47 to 1.78; *P* = 0.80)), the patients in the arm A were allowed to cross over after liver progression (2.1 versus 4.5 months in arm B, HR = 0.51; 95% CI, 0.28 to 0/94; *P* = 0.03) [23].

**Figure 5:**
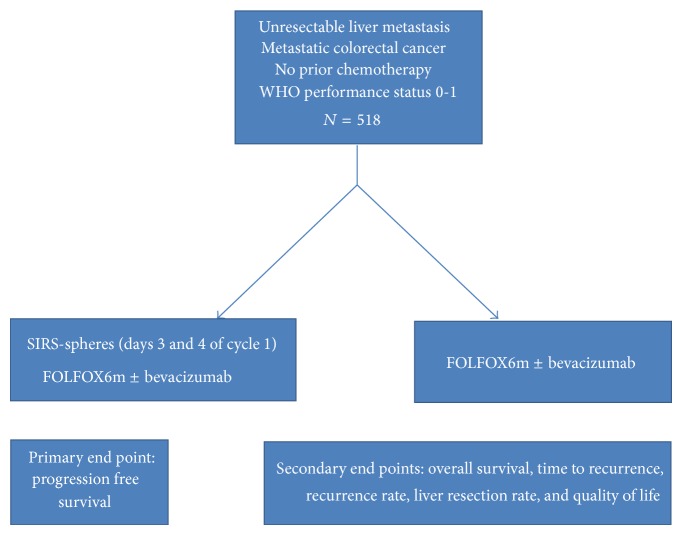
Design of the SIRFLOX study.

In summary, several trials have demonstrated a good safety and efficacy profile of radioembolization with yttrium 90 microspheres in colorectal cancer metastatic to the liver and have shown overall response rates and overall survival benefits. Gray evaluated in a phase III randomized clinical trial the role of a single dose Y90 radioembolization to hepatic artery chemotherapy, achieving better overall response rates and progression free disease, with a trend to improved median overall survival at 15 months [24]. A Y90 radioembolization trial involving FOLFOX as first-line chemotherapy is ongoing (the SIRFLOX study, Figure 5).

## Figures and Tables

**Table 1:** Chronologic assessment and therapy of patient.

Date	Chemotherapy	Biologic therapy	Y90 treatment	Chest CT	Abdomen CT	Abdominal MRI
06/07/13	—	—	—	Lung mass in the right lower lobe	Multiple liver lesions	—

06/17/13	FOLFIRI	Aflibercept	—	—	—	—

07/03/13	—	—	Right lobe radio embolization	—	—	—

7/15/13	FOLFIRI	Held due to risk of bleeding from radioembolization	—	—	—	—

08/08/13	—	—	Right and left lobe radio embolization	—	—	—

08/19/13	FOLFIRI	Aflibercept resumed	—	—	—	—

09/02/13	FOLFIRI	Aflibercept	—	—	—	—

09/20/13	FOLFIRI	Aflibercept	—	Stable pulmonary nodules	Stable liver hypodensities	Interval decrease in size of the hepatic masses in the right lobe of the liver

10/07/13	FOLFIRI	Aflibercept	—	—	—	—

10/21/13	Omission of 5-FU due to fatigue and dehydration but continued with irinotecan	Aflibercept	—	—	— —	— —

11/18/13	Irinotecan	Aflibercept held due to worsening fatigue	—	—	—	—

11/25/13	Irinotecan	Held Aflibercept due to fatigue	—	—	—	—

12/2/13	Irinotecan	Aflibercept resumed at 50% reduced dose	—	—	—	—

12/16/13	Irinotecan	Aflibercept continued at 50% reduced dose	—	—	—	—

12/30/13	Chemotherapy held due to G3 fatigue and G3 diarrhea	Chemotherapy held due to G3 fatigue and G3 diarrhea	—	Interval increase of lung nodules (Progression)	Stable disease within the liver, interval development of small abdominal ascites (from trace on prior imaging) and slightly nodular contour to the liver, reflecting treatment related effects of prior radioembolization and chemotherapy.	—

1/21/14	Started on a phase I study with a novel anti-VEGFR3 antibody	—	—	—	—	—
